# Validation of Next-Generation Sequencing of Entire Mitochondrial Genomes and the Diversity of Mitochondrial DNA Mutations in Oral Squamous Cell Carcinoma

**DOI:** 10.1371/journal.pone.0135643

**Published:** 2015-08-11

**Authors:** Anita Kloss-Brandstätter, Hansi Weissensteiner, Gertraud Erhart, Georg Schäfer, Lukas Forer, Sebastian Schönherr, Dominic Pacher, Christof Seifarth, Andrea Stöckl, Liane Fendt, Irma Sottsas, Helmut Klocker, Christian W. Huck, Michael Rasse, Florian Kronenberg, Frank R. Kloss

**Affiliations:** 1 Division of Genetic Epidemiology, Medical University of Innsbruck, Innsbruck, Austria; 2 Department of Database and Information Systems, Institute of Computer Science, Leopold-Franzens University of Innsbruck, Innsbruck, Austria; 3 Department of Urology, Medical University of Innsbruck, Innsbruck, Austria; 4 Institute of Analytical Chemistry and Radiochemistry, Leopold-Franzens University of Innsbruck, Innsbruck, Austria; 5 Department for Cranio-, Maxillofacial and Oral Surgery, Medical University of Innsbruck, Innsbruck, Austria; Kunming Institute of Zoology, Chinese Academy of Sciences, CHINA

## Abstract

**Background:**

Oral squamous cell carcinoma (OSCC) is mainly caused by smoking and alcohol abuse and shows a five-year survival rate of ~50%. We aimed to explore the variation of somatic mitochondrial DNA (mtDNA) mutations in primary oral tumors, recurrences and metastases.

**Methods:**

We performed an in-depth validation of mtDNA next-generation sequencing (NGS) on an Illumina HiSeq 2500 platform for its application to cancer tissues, with the goal to detect low-level heteroplasmies and to avoid artifacts. Therefore we genotyped the mitochondrial genome (16.6 kb) from 85 tissue samples (tumors, recurrences, resection edges, metastases and blood) collected from 28 prospectively recruited OSCC patients applying both Sanger sequencing and high-coverage NGS (~35,000 reads per base).

**Results:**

We observed a strong correlation between Sanger sequencing and NGS in estimating the mixture ratio of heteroplasmies (r = 0.99; p<0.001). Non-synonymous heteroplasmic variants were enriched among cancerous tissues. The proportions of somatic and inherited variants in a given gene region were strongly correlated (r = 0.85; p<0.001). Half of the patients shared mutations between benign and cancerous tissue samples. Low level heteroplasmies (<10%) were more frequent in benign samples compared to tumor samples, where heteroplasmies >10% were predominant. Four out of six patients who developed a local tumor recurrence showed mutations in the recurrence that had also been observed in the primary tumor. Three out of five patients, who had tumor metastases in the lymph nodes of their necks, shared mtDNA mutations between primary tumors and lymph node metastases. The percentage of mutation heteroplasmy increased from the primary tumor to lymph node metastases.

**Conclusions:**

We conclude that Sanger sequencing is valid for heteroplasmy quantification for heteroplasmies ≥10% and that NGS is capable of reliably detecting and quantifying heteroplasmies down to the 1%-level. The finding of shared mutations between primary tumors, recurrences and metastasis indicates a clonal origin of malignant cells in oral cancer.

## Introduction

Squamous cell carcinoma of the oral cavity (OSCC) account for 95% of all malignant lesions of the mouth and have become almost synonymous with oral cancer [1]. The development of OSCC is a multistep process modulated by genetic predisposition, chronic inflammation, tobacco and alcohol abuse and viral infections typically acquired via oral-genital contact [1, 2]. The overall five-year survival rate of OSCC is ~50% and has not changed significantly during the past 30 years [3, 4]. Therapeutic decisions are usually based on clinical and histopathological parameters, which however often fail to predict patient outcome and therapy success. Therefore, the search for new prognostic and predictive factors of OSCC reflects the need for improved risk assessment to customize therapeutic approaches.

Mitochondria have been implicated in the process of carcinogenesis because of their vital role in energy production [5], control of metabolic pathways [6] and the high mutation rate of their DNA (mtDNA) [7]. The most important aspect for medical mtDNA research is the occurrence of heteroplasmy, which is defined as the coexistence of two or more populations of mtDNA molecules with slightly different nucleotide composition in a single mitochondrion, cell, tissue or individual. Heteroplasmic mtDNA mutations were found in normal human cells [8–11] as well as in many human tumors [12–15].

Previously, our group could demonstrate that prostate tumor-specific mtDNA heteroplasmies were associated with elevated PSA-levels and might thus allow early detection of prostate cancer [14]. Recent studies on the involvement of mtDNA mutation in OSCC suggest that pathogenic mtDNA heteroplasmies are potential prognostic markers for OSCC [16]. The detection of low levels of heteroplasmy in tumors may allow unprecedented early identification and monitoring of neoplastic progression to malignancy. Therefore, a sensitive detection and accurate quantification of heteroplasmic changes may assist in a personalized risk assessment.

Conventional Sanger sequencing is not sensitive enough to detect low level heteroplasmy, i.e. heteroplasmy <10%. In addition, the ratio of peak heights at heterozygous positions or any other sequence variant are context-dependent and may differ strongly between forward and reverse strands [17]. However, NGS on an Illumina HiSeq offers a robust platform for comprehensive mtDNA analysis, as the small size of the mitochondrial genome and the resulting high coverage for each nucleotide position enable a more sensitive and accurate quantification of low-level heteroplasmy [18]. Currently, we are in the midst of a transition process from Sanger sequencing to NGS-based molecular diagnosis of mtDNA disorders, and several studies have already demonstrated that NGS on an Illumina platform is sensitive and accurate in detecting heteroplasmic mtDNA changes [15, 19–22].

The purpose of this research project was to evaluate the application of next-generation sequencing on an Illumina HiSeq platform for detecting and quantifying heteroplasmic mixtures in cancer tissue samples. The second goal was to explore the variation of somatic mtDNA mutations in primary oral carcinomas, recurrences and metastases.

Twenty-eight oral cancer patients were prospectively enrolled and followed for a median of 30.5 months. The entire mitochondrial genome of 85 tissue samples (primary tumors, recurrences, resection margins, blood, lymph node metastases, one dysplasia and one second primary tumor) was genotyped in 91% of cases with both Sanger and next-generation sequencing, except for a few samples, where not enough DNA was available for both sequencing procedures. The combined evaluation of clinical findings, histopathological analyses and of the results from the genetic analyses of different tissues enabled an assessment of the efficiency of low-level mtDNA mutation screening.

## Results

### Determination of the lower detection limit of heteroplasmies

**Fig 1:**
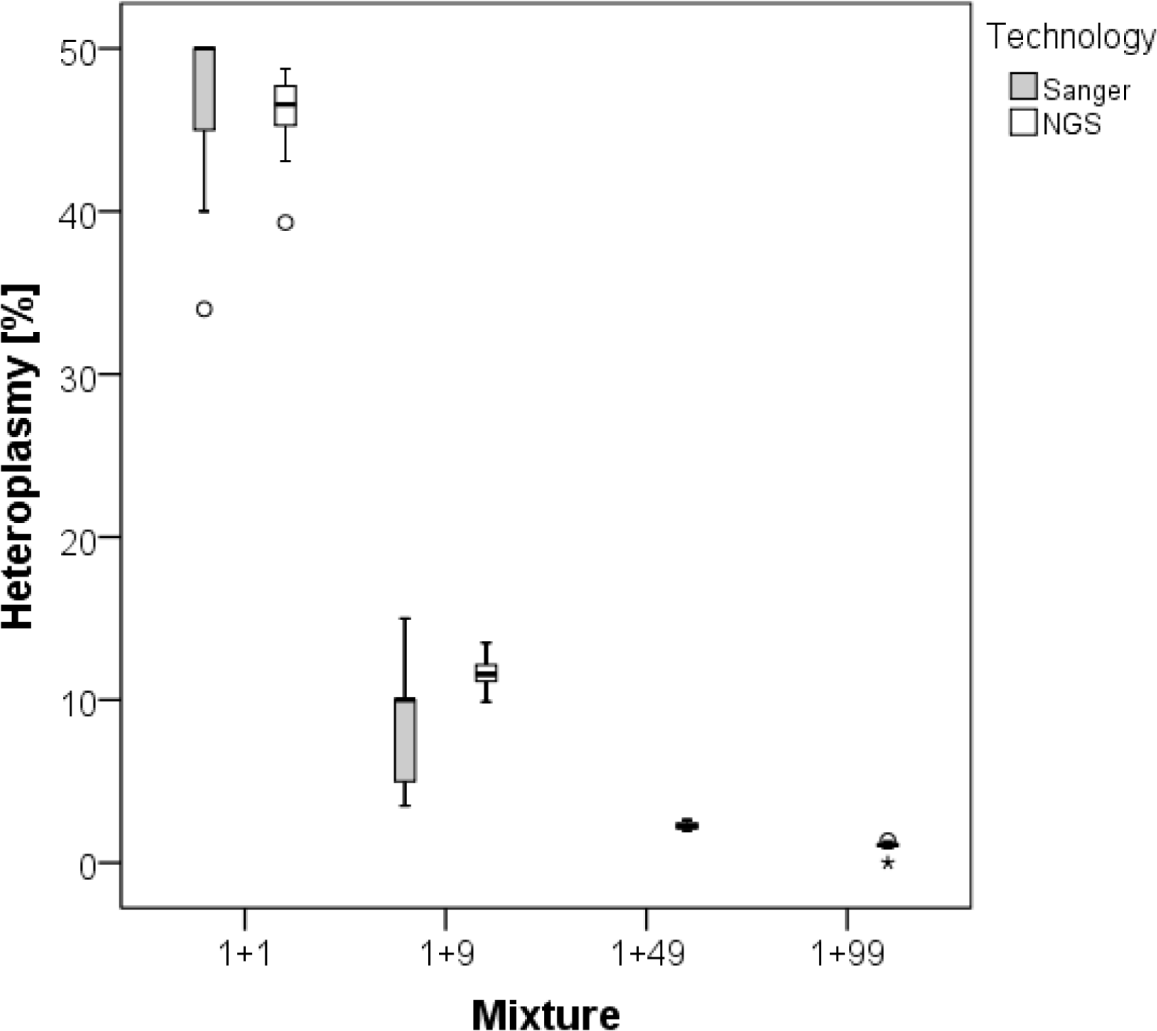
Percentage of the minor component on heteroplasmic positions as obtained with Sanger sequencing and next-generation sequencing on an Illumina HiSeq platform. The samples contained pre-defined mixtures of the DNA of two lab members. In Sanger electropherograms, heteroplasmies were neither detected in the 1+49 mixture nor in the 1+99 mixture.

In order to determine the lower detection limit of heteroplasmies, four samples with pre-defined mixtures of the DNA of two lab technicians (Lab002 and Lab011) with mixture ratios of 1+1, 1+9, 1+49 and 1+99 were subjected to both Sanger [14] and next-generation sequencing. The two technicians differed on 27 nucleotide positions dispersed over the entire genome. Despite the high sequence coverage and the excellent signal-to-noise ratios in the sequence electropherograms from Sanger sequencing, only heteroplasmic mixtures at the 7% level could be detected (**Fig 1**). For NGS, the samples were sequenced on an Illumina HiSeq 2500 platform. With the high NGS coverage for the four mixture sample as well as the two original DNA samples, we were able to exactly quantify the minor components at the 1% level as obtained from the 1+99 mixture by lowering the detection threshold to 0.8% within the mtDNA-Server application. Position 16189 was not found at the 1% level. However, we found three additional mutations at 9462, 15236 and 16129, which were confirmed as private heteroplasmic mutations from the original DNA sample Lab01. These mutations however were true and cannot be regarded as false positives, as they were detected in the original DNA sample and in the 1+1-mixture. All results from the mixture experiment are given in **S4 Table**.

### Comparison between Sanger and next-generation sequencing

Similar to a study on nuclear DNA mutations [23], the limit of detection for Sanger sequencing was identified at 7% of mutant mtDNA to the reference genome rCRS [24]. As all samples from our study were subjected to both Sanger and NGS on an Illumina platform, we compared the estimates of the percentages of the minor components in point heteroplasmies between Sanger electropherograms (as calculated with Sequencher) and NGS reads for all point heteroplasmies and found a very strong correlation between the two measurements (Pearson’s r = 0.99; p<0.001). For heteroplasmies <10%, which were detected with NGS, but could not be found in Sanger electropherograms, because they could not be distinguished from the sequence background, the value for the Sanger estimation was set to $\frac{10}{\sqrt{2}}$, corresponding to $Limit\;of\;detection/\sqrt{2}$ as suggested Croghan and Egehy [25].

**Fig 2:**
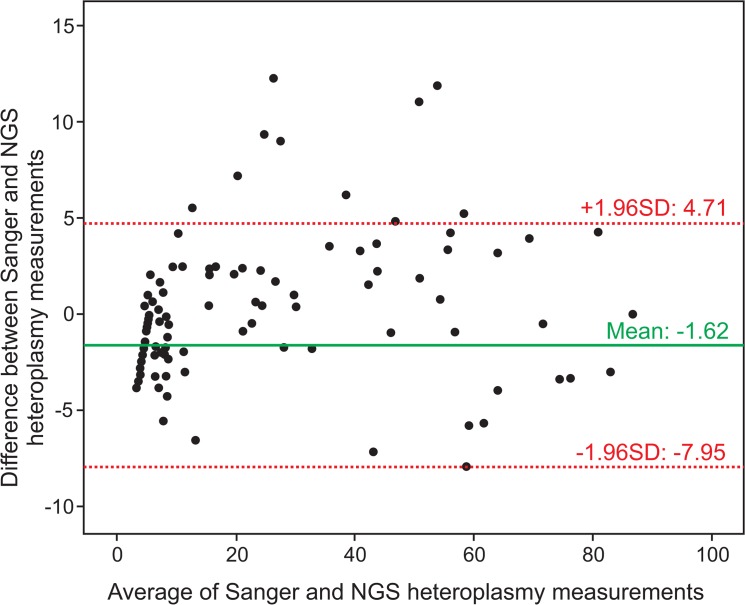
Bland-Altman plot for depicting the agreement between NGS and Sanger sequencing heteroplasmy measurements. On the x-axis, the average of next-generation and Sanger sequencing heteroplasmy estimates is plotted. On the y-axis, the difference between next-generation and Sanger sequencing heteroplasmy estimates is plotted. The mean difference is indicated as green line, the 95% limits of agreement (average difference ± 1.96 standard deviation of the difference) are indicated as red dotted lines. For heteroplasmies 10%, which were detected with NGS, but could not be found in Sanger electropherograms, the value for the Sanger estimation was set to $\frac{10}{\sqrt{2}}$.

However, a high correlation for any two methods designed to measure the same property does not automatically imply that there is good agreement between the two methods [26]. Therefore, a Bland-Altman plot was created for analyzing the agreement between Sanger sequencing and NGS heteroplasmy measurements (**Fig 2**). All but ten values lay within the 95% limits of agreement. The mean value of the difference (NGS–Sanger = -1.62) between the two measurements was significantly different from 0 (p<0.001), indicating that there was a systematic difference between the two methods, with Sanger sequencing slightly underestimating the heteroplasmy level. However, as that 95.3% of values lay within the 95% limits of agreement, we concluded that NGS and Sanger sequencing can be used interchangeably for determining the mixture ratios on point heteroplasmic positions, where the minor component amounted for at least 10% of the nucleotide mixture.

### Mitochondrial DNA sequences

**Fig 3:**
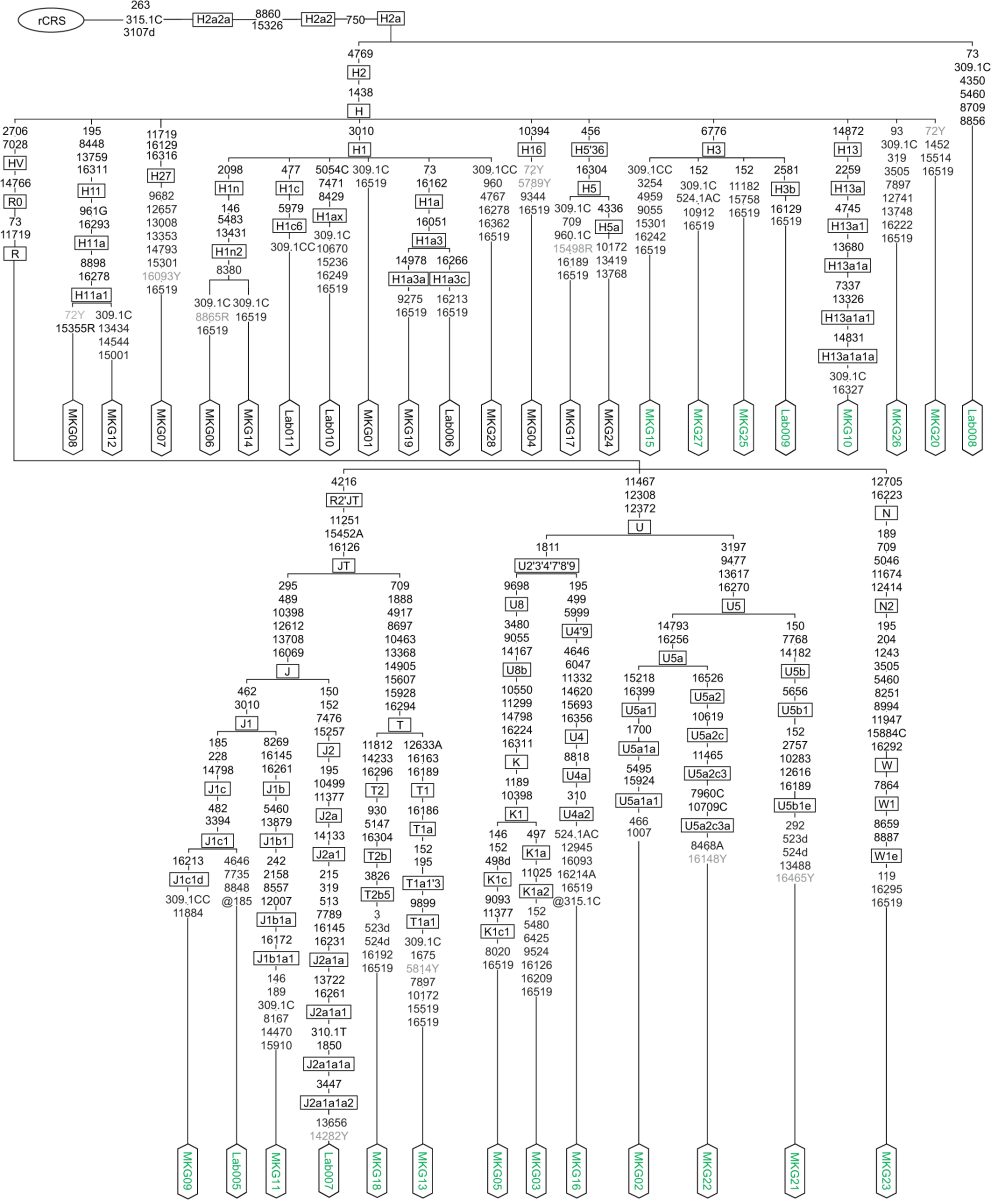
Phylogenetic tree representing the benign profiles of 28 oral cancer patients and seven team members. The sequences are stored in GenBank (accession numbers KC286583 –KC286589 corresponding to Lab005 –Lab011 and KC286590 –KC286617 corresponding to MKG01 –MKG28). The position of the rCRS is indicated for reading off sequence motifs. Mutations are transitions unless a base is explicitly indicated. The prefix ‘‘@” designates reversions, whereas suffixes indicate transversions (to A, G, C, or T), indels (.1, d), and heteroplasmies (R, Y). Point heteroplasmic mutations that were observed in benign tissue samples are highlighted in grey.

The entire mitochondrial genome was sequenced using both Sanger and Illumina HiSeq next-generation sequencing from 77 of the 85 tissue samples (primary tumors, recurrences, resection margins of both primary tumors and recurrences, blood, cancerous and benign areas in lymph nodes, one dysplasia and one second primary tumor) (**S1 Table**) collected from 28 prospectively enrolled oral cancer patients (**Fig 3**). Only from 8 samples (6 paraffin samples, two benign tissue samples) we could not extract enough DNA to subject them to both sequencing technologies, so they were only typed with Sanger sequencing (**S2 Table**). The sequences from benign tissue samples were stored in GenBank (accession numbers KC286583 –KC286589 corresponding to Lab005 –Lab011 and KC286590 –KC286617 corresponding to MKG01 –MKG28).

### Distribution of inherited and somatic variations across genomic regions

All observed point and length heteroplasmic mutations are described in **S2 Table**. Length heteroplasmy occured frequently within the polycytosine stretches of HVS-I (anywhere between positions 16188 and 16195) and HVS-II (around position 310) due to replication slippage when a specific minimum number of cytosine residues are present [8, 14]. Interestingly, tumor samples showed a higher tendency to exhibit length heteroplasmy than their corresponding benign tissue samples. Four tumor samples (MKG01, MKG10, MKG12 and MKG26) showed additional cytosine-insertions in the HVS-II C-stretch after position 309 compared to their benign tissue samples, while only one tumor sample (MKG11) showed fewer C-residues in the HVS-II C-stretch with respect to its benign tissue sample. One tumor sample (MKG13) showed length heteroplasmy after position 573 in HVS-III, but its corresponding benign sample did not show any signs of heteroplasmy at this position. The most interesting observation was that two unrelated tumor samples (MKG11 and MKG21) exhibited frame-shift causing length heteroplasmy in MT-ND5 on position 12390, which was not seen in the associated benign samples.

All OSCC patients showed somatic mtDNA alterations in their index tumors and/or benign tissue samples. However, when considering Sanger sequencing data only, then tumor-specific mtDNA heteroplasmies could only be detected in 20 out of 28 patients. We observed cancer-specific variants (primary tumors, recurrences and lymph node metastasis) on 124 different nucleotide positions. Homoplasmic polymorphisms that were detected in all tissue samples of a patient were regarded as germline variants. Most somatic variations were singletons, arising in only one patient. In total, 6.9% of somatic mutations were within tRNAs, 18.3% within ribosomal RNAs, 52.2% in protein-coding regions, and 22.6% in the control region.

There was a strong correlation between the proportion of somatic variants and the proportion of inherited variants in a given gene region (r = 0.85; p = 3.7x10^-5^), and among protein-coding genes, both the proportion of somatic (r = 0.81; p = 0.001) and inherited nucleotide variations (r = 0.74; p = 0.005) correlated with the proportional length of the gene in relation to the entire mitochondrial genome (**S1 Fig**). In addition, there was a significant enrichment of non-synonymous variants among cancerous tissue samples as compared with germline variants (p<0.001).

### Comparison with other NGS-typed oral cancer mitochondrial genomes

**Fig 4:**
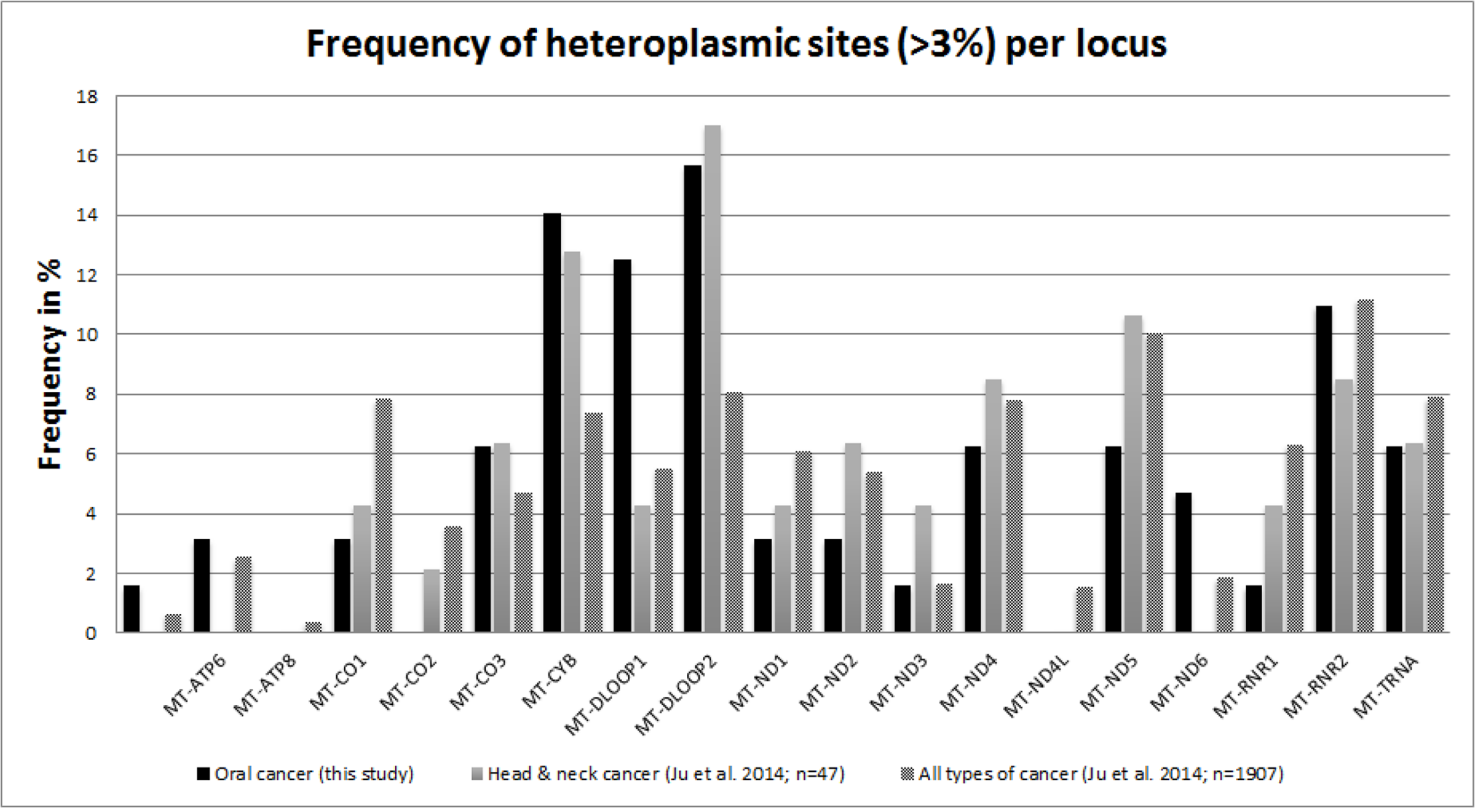
Distribution of NGS-typed heteroplasmies across the mitochondrial genome. Frequencies were calculated as number of heteroplasmies per gene region divided by the total number of observed heteroplasmies. The data from our study were compared with data from head and neck cancers and with all cancer samples from Ju et al. [27].

We were interested whether the distribution of heteroplasmies across genetic regions as observed in our study could be characteristic for other oral cancer mitochondrial genomes, as this would corroborate the authenticity of the variations that we found. Therefore, we compared the frequency distribution of heteroplasmic sites found in our tumor samples with the frequency distribution of heteroplasmies detected in 47 head and neck tumor samples and with 1907 samples of diverse carcinomas [27] derived from whole genome and whole exome sequencing. Since Ju et al. applied a 3% filter for the variant allele frequency, we restricted our data specifically for this comparative analysis to heteroplasmies >3%. The dispersion of heteroplasmies in our sample was highly correlated with the heteroplasmy distribution in head and neck tumors (r = 0.79; p<0.001) and with the heteroplasmy allocation in diverse tumor samples (r = 0.62; p<0.001) (**Fig 4**). Therefore, the frequency of heteroplasmies in diverse gene regions found in our study seems to reflect well the distinct vulnerability of diverse areas of the mitochondrial genome to cancerous somatic mutations.

### Heteroplasmic mutations shared between benign and cancerous tissue samples

One recent study suggested that many putative somatic mutations are in fact low-level heteroplasmies undetected in the germline that have undergone clonal expansion in the tumor [28]. In our study, 54% of the patients shared mutations between benign and cancerous tissue samples (MKG01: m.G14560R; MKG04: m.T5789Y; MKG05: m.C16261Y; MKG06: m.G8865R; MKG07: m.T16093Y; MKG08: m.G15355R; MKG11: m.T146Y; MKG13: m.T5814Y and m.C13287Y; MKG15: m.A9794R; MKG17: m.G15498A; MKG21: m.G13759R and m.C16465Y; MKG22: m.C16148Y; MKG23: m.T4597Y; MKG27: m.A215R, m.A9702R and m.A16241R; MKG28: m.C64Y).

In MKG04 (m.T5789Y), MKG06 (m.G8865R) and MKG08 (m.G15355R), the heteroplasmies were also found in the blood samples of the patients, underscoring that those were in fact germline mutations. However, we only analyzed blood samples from 4 out of 28 patients (14.3%), and in those, we found consistencies between tumor and blood heteroplasmies in 3 out of 4 patients (75%).

### Heteroplasmic mutations shared between different areas of the same tissue sample

For three patients, distinct areas within the same tumor entity were used for microdissection, because these areas differed from each other from a histopathologically perspective. In patient MKG01, two separate cancerous areas were sequenced, which both shared exactly the same three mutations (m.G14560A, m.T15033C and m.G15553A). This observation was in contrast to patient MKG03, where two separate tumor areas showed a completely different mutation profile (primary tumor area 1: m.G5881A, m.T11790C, m.T14787C and m.G15106A; primary tumor area 2: m.T10983C). For patient MKG27, two distinct benign areas were sampled, which shared one heteroplasmic mutation (m.A16241G), but also differed on four other positions (benign area 1: m.A3523G and m.A14002G; benign area 2: m.A215G and m.A9702G). In addition, three distinct tumor areas were sequenced, which shared only one mutation (m.A215G). Primary tumor area 2 shared another mutation with primary tumor area 1 (m.G2690A), and primary tumor area 1 shared four mutations with primary tumor area 3 (m.A183G, m.A9702G, m.T13897C and m.A16241G), which showed on mutation that was not observed in any other benign or cancerous area (m.A1082G). To sum up, in samples where different areas within the same tumor were selected for DNA extraction and mtDNA sequencing, we observed both concordances and discordances in the mutational pattern of mtDNA heteroplasmies.

### Somatic mutations in recurrences

**Fig 5:**
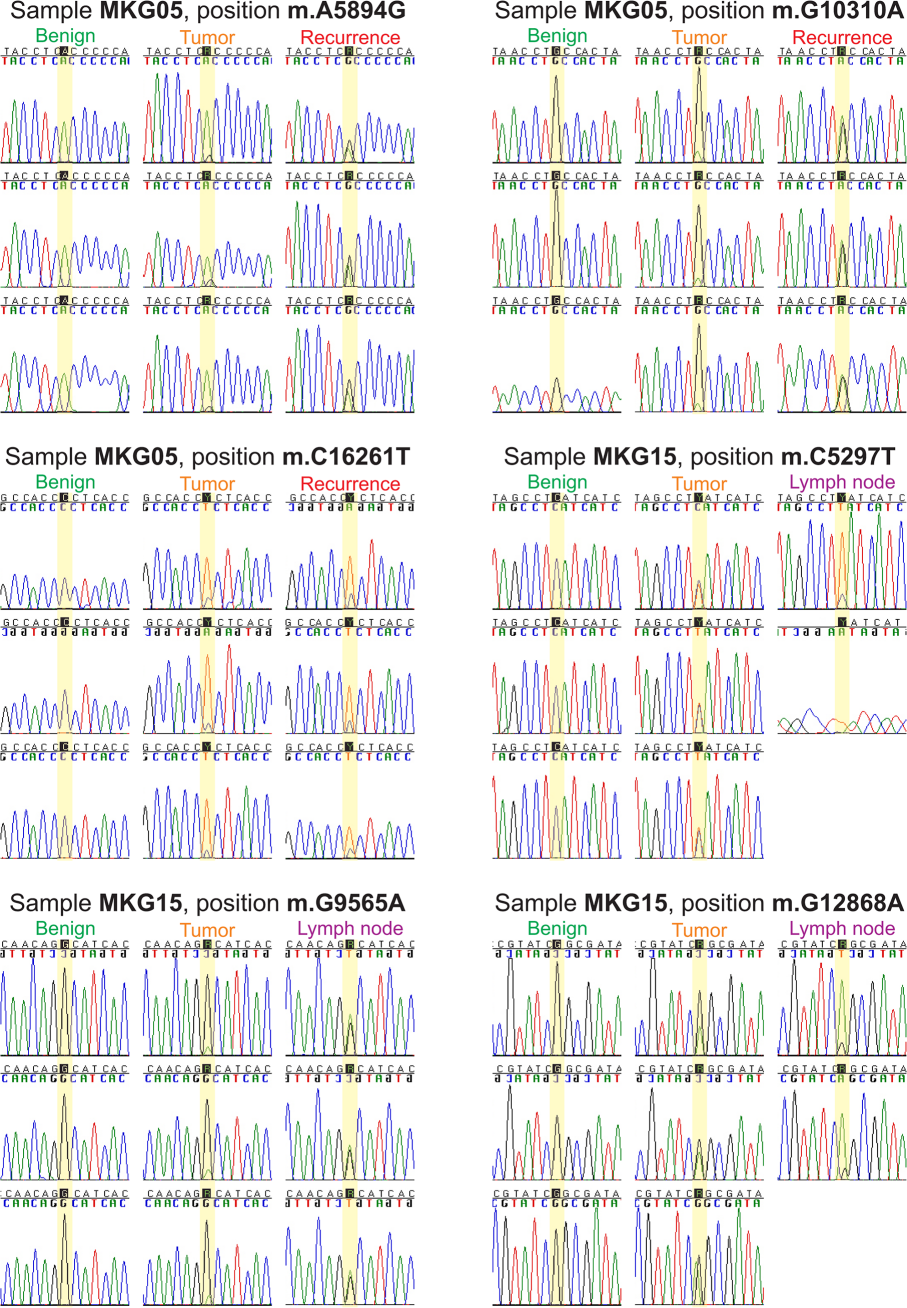
Heteroplasmic mutations as seen with Sanger sequencing in MKG05 and MKG15. The benign tissue was taken from the resection border of the tumor.

**Fig 6:**
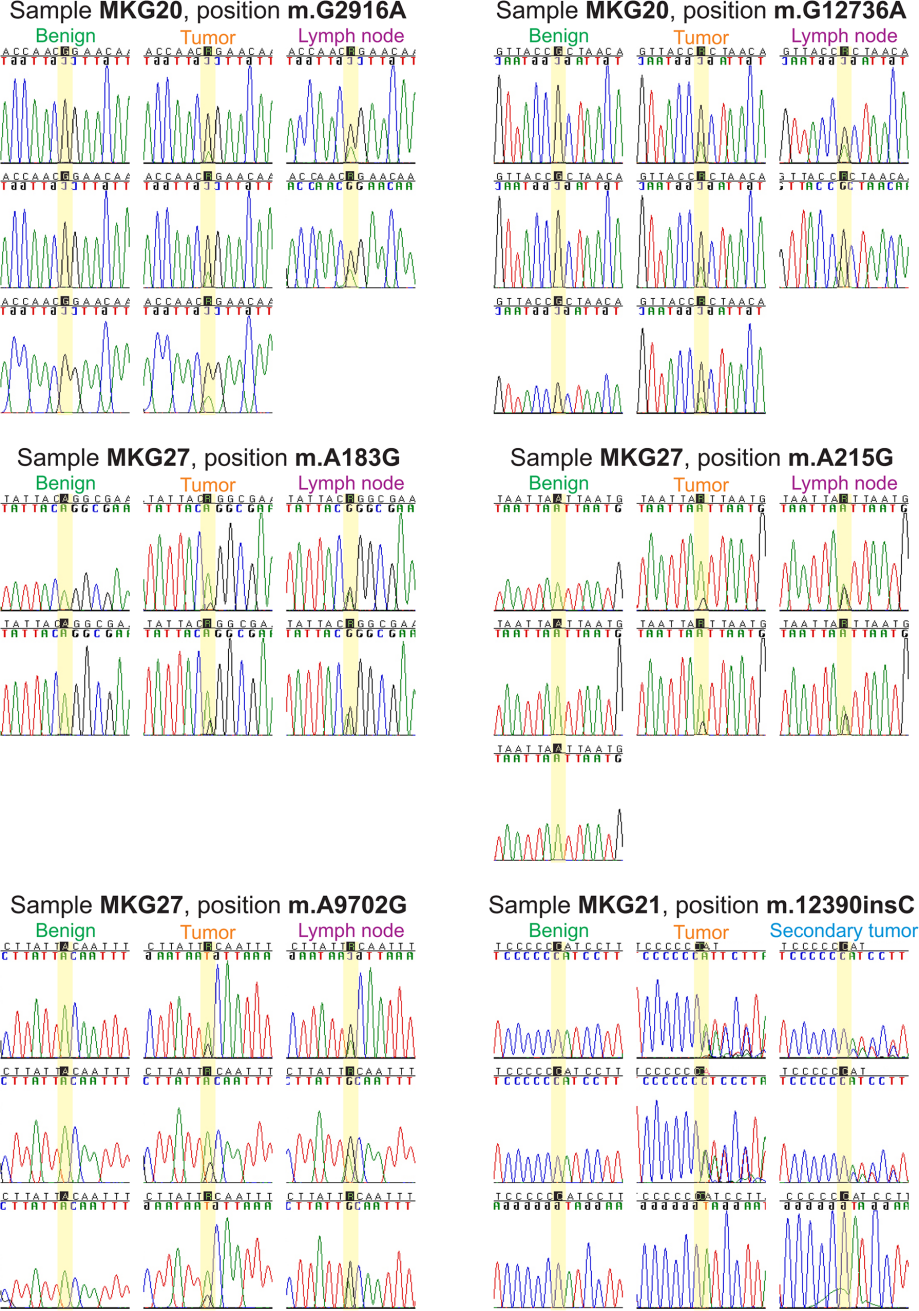
Heteroplasmic mutations as seen with Sanger sequencing in MKG20, MKG21 and MKG27. The benign tissue was taken from the resection border of the tumor.

Six patients developed a tumor recurrence (MKG05, MKG10, MKG13, MKG21, MKG24 and MKG27) during follow-up, four of these patients (MKG05, MKG10, MKG13 and MKG21) showed mutations in the recurrence that had also been observed in the index tumor and in three of these patients (MKG05, MKG13 and MKG21), the mutations were also observed in the resection margins (**Table 1**). On three out of five nucleotide positions, patient MKG05 showed an increase of the proportions of the mutated nucleotides from the index tumor (~13%) to the recurrence (~70%). However, on two nucleotide positions in the mtDNA profile of patient MKG05, we observed an average decrease of ~9% from index tumor heteroplasmies to recurrence heteroplasmies (**Tables 1**and **2**and **Fig 5**). Patient MKG21 was first surgically treated because of OSCC in the mandible, and 16 months later a second OSCC was removed from this patient’s maxilla. The most intriguing observation was that the mandibular tumor showed length heteroplasmy after position 12390, which was also found in the maxillary tumor, but not in the benign area surrounding the first tumor (**Fig 6**and **S2 Table**).

**Table 1:** Occurrence of somatic mtDNA mutations in different tissues of the same patient.

Patient	Mutation	Benign	Blood	Primary tumor	Recurrence	Tumor in lymph node
MKG05	C64Y	n.d.	n.d.	12.13	62.3	n.a.
MKG05	A5894R	n.d.	n.d.	15.54	71.6	n.a.
MKG05	G6762R	n.d.	n.d.	7.14	1.2	n.a.
MKG05	G10310R	n.d.	n.d.	10.8	61.2	n.a.
MKG10	T9865Y	n.d.	n.a.	1.76	1.6	n.a.
MKG15	C5297Y	n.d.	n.a.	45.87	n.a.	84.8
MKG15	G9565R	n.d.	n.a.	12.41	n.a.	55.1
MKG15	G12868R	n.d.	n.a.	37.8	n.a.	85.8
MKG20	G2916R	n.d.	n.a.	21.02	n.a.	31.5
MKG20	G12736R	n.d.	n.a.	24.73	n.a.	32
MKG27	A183R	n.d.	n.a.	22.7	n.a.	57.5
MKG27	T13897Y	n.d.	n.a.	2.4	n.a.	1.9

Notes: The tables indicate the percentage of the mutated nucleotide (relative to the rCRS) on point-heteroplasmic positions. Only mutations, which occurred in more than one tissue sample of a patient, were included in these tables.

n.a. tissue was not available

n.d. respective heteroplasmy was not detected above the threshold of 1%

**Table 2:** Occurrence of shared mtDNA mutations considered “germline mutations”

Patient	Mutation	Benign	Blood	Primary tumor	Recurrence	Tumor in lymph node
MKG01	G14560R	1.65	n.a.	1.38	n.a.	n.a.
MKG04	T5789Y	16.91	45.62	8.21	n.a.	n.a.
MKG05	C16261Y	1.27	n.d.	87.02	74.8	n.a.
MKG06	G8865R	27.74	20.86	65.87	n.a.	n.a.
MKG07	T16093Y	22.39	n.a.	10.58	n.a.	n.a.
MKG08	G15355R	52.08	56.66	10.37	n.a.	n.a.
MKG11	T146Y	98.73	45.16	n.a.	n.a.	n.a.
MKG13	C13287Y	3.53	n.a.	1.38	1.7	n.a.
MKG13	T5814Y	6.73	n.a.	4.15	5.3	n.a.
MKG15	A9794R	2.84	n.a.	1.63	n.a.	74.7
MKG17	G15498R	23.73	n.a.	30.5	n.a.	n.a.
MKG21	G13759R	2.66	n.a.	1.26	1.54	n.a.
MKG21	C16465Y	89.32	n.a.	41.85	43.35	n.a.
MKG22	C16148Y	30.5	n.a.	6.17	n.a.	n.a.
MKG23	T4597Y	1.28	n.a.	1.3	n.a.	n.a.
MKG27	A16241R	1.1	n.a.	1.14	n.a.	n.d.
MKG27	A9702R	8.6	n.a.	25.4	n.a.	58.9
MKG27	A215R	24	n.a.	32.5	n.a.	56.7
MKG28	C64Y	6.83	n.a.	3.33	n.a.	n.a.

Notes: The tables indicate the percentage of the mutated nucleotide (relative to the rCRS) on point-heteroplasmic positions. Only mutations, which occurred in more than one tissue sample of a patient, were included in these tables. The recurrence in patient MKG21 should be regarded as second primary tumor, as the primary oral squamous cell carcinoma (OSCC) of this patient occurred in her mandible, while the second OSCC was found in her maxilla.

n.a. tissue was not available

n.d. respective heteroplasmy was not detected above the threshold of 1%

Patient MKG27 developed a recurrence, but died before a potential second surgery. Therefore, we did not have a tissue sample from the recurrence available for analysis. However, similar to patients MKG05, MKG13 and MKG21, we found two of the somatic tumor mutations also in the benign tissue surrounding the index tumor of patient MKG27 (**Table 2**). Eleven further patients (MKG01, MKG04, MKG06, MKG07, MKG08, MKG11, MKG15, MKG17, MKG22, MKG23 and MKG28) shared mutations between tumor areas and benign areas surrounding the tumor (**S2 Table**), but did not develop a recurrence during follow-up.

Those somatic mutations, i.e. mutations that were not present in the benign tissue, which were also found in either the recurrence or the lymph node metastases, amounted for on average 17.9% of the mixture, and increased to an average of 39.6% in recurrences and to an average of 49.8% in lymph node metastases (**Table 1**). The average heteroplasmy level as computed from **S6 Table** was 16.4%. Therefore, we saw no difference to the mean value of heteroplasmies in cancerous samples from the entire data set. However, it was interesting to see that 75% of somatic mutations, which were shared between tissues, were high-level (i.e. larger 10%), while in **S6 Table**, only 36.1% of somatic mutations in cancer tissues were high-level. The observation that higher level heteroplasmies were more likely to be shared could be yield further evidence for the clone expansion hypothesis.

In summary, 83% of the recurrences shared heteroplasmic mutations with the primary tumor, and 50% of the recurrences also shared heteroplasmic mutations with the resection margins of the primary tumor. However, the sharing of heteroplasmic mutations between primary tumors and resection margins was not associated with tumor relapse in our study (p>0.05).

### Somatic mutations in lymph nodes

**Fig 7:**
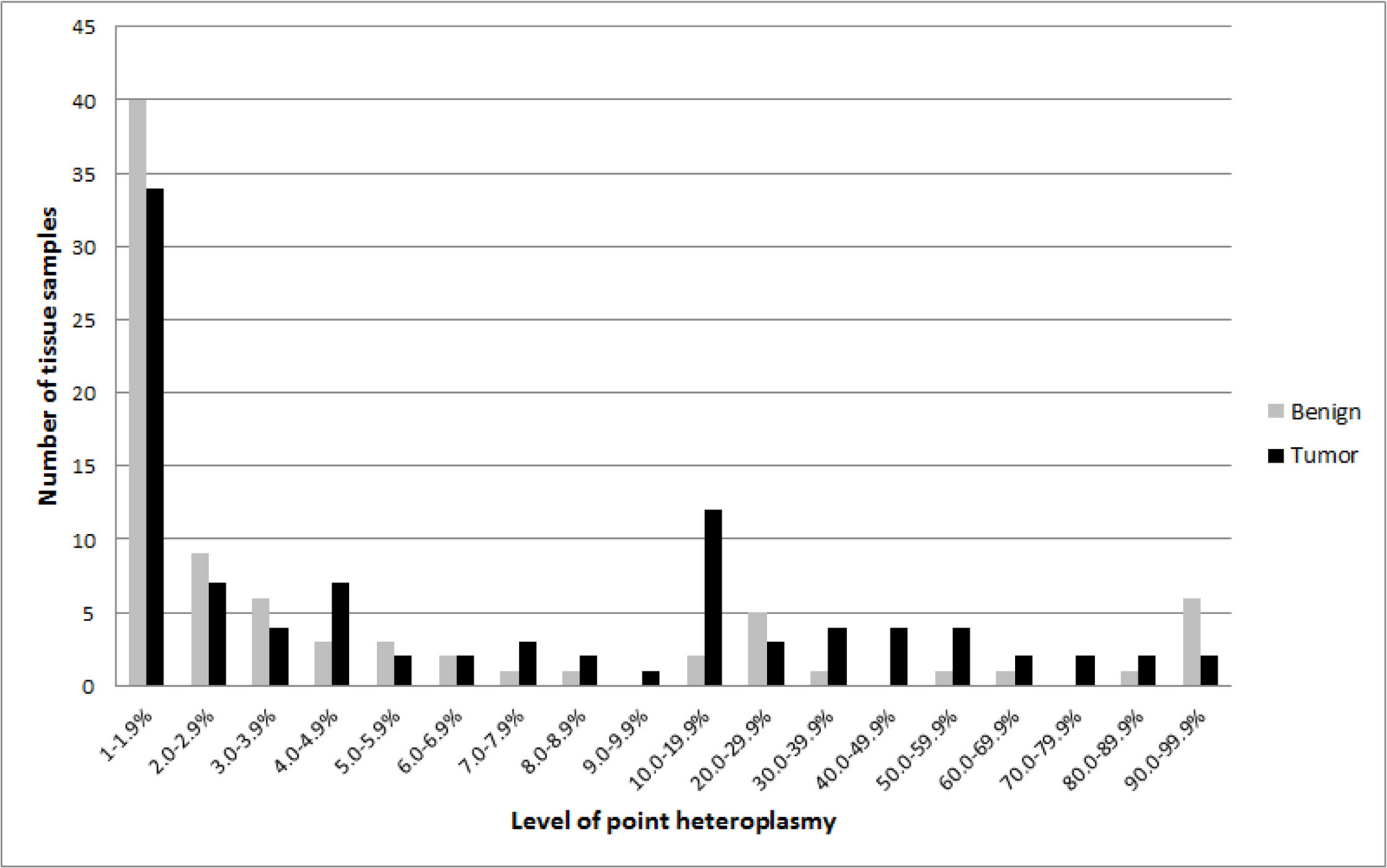
Distribution of point heteroplasmy levels in tumor and benign tissue samples.

Five patients showed tumor metastases in the lymph nodes of their necks (MKG15, MKG18, MKG20, MKG26 and MKG27). Three of these patients (MKG15, MKG20 and MKG27) shared mtDNA mutations between index tumors and lymph node metastases (**Table 1**). In MKG26, the primary tumor showed only one somatic mutation, which was not found in the lymph node metastasis, which itself harbored six somatic mtDNA heteroplasmies. A consistent increase of the proportions of mutated nucleotides on all but one shared heteroplasmic sites from the oral carcinoma to the lymph node metastases was observed in all three patients (MKG15: from ~25% to ~70%; MKG20: from ~23% to ~32%; MKG27: from ~10% to ~44%) (**Figs 6**and 7). This corresponded to an increase by a factor of 2.8 in MKG15, by a factor of 1.4 in MKG20, and by a factor of 4.2 in MKG27. The patients died rapidly (7–15 months) after tumor resection.

### Comparison of heteroplasmy between tumor and benign tissue samples

When comparing heteroplasmy levels between tumor and benign tissue samples, it was obvious that low level heteroplasmies, defined as heteroplasmies <10%, were more frequent in benign compared to cancerous tissue samples, while heteroplasmies with minor alleles above 10% dominated in tumor samples (p<0.001; **Fig** 7). Indeed, 65 out of 82 (79.3%) heteroplasmic mutations found in benign tissues were below 10% (**S5 Table**), while 62 out of 97 (63.9%) of heteroplasmic mutation found in tumor tissues were below 10% (**S6 Table**; Chi-Square-Test: p = 0.024). However, when comparing the phylogenetic stability of the mutations between the different tissue types, there was no statistical evidence that the average phylogenetic weights as estimated with HaploGrep [29] or EMMA [30] differed between the four main tissue groups (benign, primary tumor, recurrence, metastases) (p>0.05). Therefore, we found no evidence for a preference of mutating phylogenetically stable and thus probably physiologically important sites in tumor samples. However, given our sample size, we also lack statistical power for such a comparison.

### Exclusion of potential next-generation sequencing artifacts

In order to exclude phantom mutations, which are systematic artifacts generated in the course of the sequencing process, the frequencies of heteroplasmic sites were inspected closely. Two positions, namely 414K and 72Y showed up in 12 and 11 different samples, respectively. It is suspicious that both positions share the same sequence neighborhood, namely GG[T]ATGCAC. This is similar to a previous description of phantom mutation hotspots in Sanger electropherograms, where a certain nucleotide motif was found to be responsible for most artifacts in single-stranded sequences [31]. In addition, the coverage on the forward reads fell compared to the neighboring bases to ~52% on 72Y and ~73% on 414K, independently if BAQ [32] was applied or not. The reverse reads did not show any reduction, but a constant coverage at the specific positions. Furthermore, some samples showed 72K on the forward reads, but 72Y on the reverse reads. We hypothesized that low-level (<5%) heteroplasmies on these positions, which were not seen in Sanger electropherograms, could be considered as potential phantom mutations. This should however be verified for example with cloning and mini-sequencing [33] or droplet digital PCR. In addition, a recent study by Li and colleagues also observed a similar pattern on position 72 [34]. Unfortunately, while many other mutations were validated with droplet digital PCR in the study by Li et al., the 72Y was not further confirmed. Still, three samples from our study showed high levels of heteroplasmy at position 72 (MKG04 benign: 60.18%, MKG08 benign: 16.62% and MKG20 benign: 27.41%), that could be verified with Sanger sequencing. We therefore excluded eight (seven benign and one tumor sample) of the low-level heteroplasmic sites at 72Y, as well as 12 occurrences of T414K, which showed a mean heteroplasmic level of 1.5%. Samples with higher heteroplasmic levels at 72Y (MKG4 benign, MKG8 benign and MKG20 benign) all showed 73R and some of these samples also showed 74K (MKG4 benign and MKG20 benign), which levels were directly correlated with the occurrence of 72Y (**S7 Table**). We therefore excluded 73R and 74K. We further deleted apparent mutations surrounding long-range PCR primer annealing sites around position 2,600, where we found unusual patterns. This was the case in samples MKG1 benign (2523–2667), MKG1 cancer (nps 2523–2667) and MKG5 benign (nps 2523–2667; please see **S3 Table** for unfiltered results). We also excluded site 3Y found in Sample 18, which is a homplasmic mutation according Sanger Sequencing and could be regard as the result of mapping problems, due to the circular nature of the mitochondrial genome.

## Discussion

To the best of our knowledge, our study is the first investigation that provides a profound comparison of Sanger and next-generation sequencing technologies applied to entire mitochondrial genomes in cancer tissue samples. All samples were subjected to both high-quality Sanger sequencing (96 600bp-electropherograms per genome) and high-coverage NGS on an Illumina HiSeq platform (~35,000 reads per base). We observed a very strong correlation between the two methods in estimating the exact mixture ratio (Pearson’s r = 0.99; p<0.001). However, the Bland-Altman plot indicated that there was a small systematic difference between the measurements, with Sanger sequencing slightly underestimating the heteroplasmy levels. Still, as 95.3% of samples lay within the 95% limits of agreement, we conclude that the two methods can be used almost interchangeably for determining the mixture ratio on point heteroplasmic positions, where the minor component amounts for at least 10% of the nucleotide mixture. Nonetheless, it has to be said that the majority of heteroplasmies lay below the detection limit of Sanger sequencing. In addition, the detection and interpretation of point heteroplasmies in Sanger electropherograms strongly depends on the experience and expertise of the evaluating mtDNA scientist, and could easily vary between different evaluations. On the contrary, the procedure of heteroplasmy detection and quantification with NGS is completely independent from the evaluator and therefore an impartial and replicable process. The reliable detection of length heteroplasmies can be considered as strength of Sanger sequencing, however we did not evaluate NGS data regarding length heteroplasmies. The major advantage of Illumina HiSeq NGS is the exact quantification of nucleotide mixtures down to the 1%-level, an observation that is in agreement with recent data [35].

When taking all available information on point heteroplasmies in our dataset into consideration (including low level variants down to 1% nucleotide mixture ratio), we found a strong correlation between the proportion of somatic variants and the proportion of inherited variants in a given gene region (r = 0.85; p = 3.7x10^-5^), indicating that certain regions of the mitochondrial genome are more susceptible to mutations in general compared to other regions. However, this observation could also be explained by the fact that among protein-coding genes, both the proportional number of somatic (r = 0.81; p = 0.001) and inherited nucleotide variations (r = 0.74; p = 0.005) correlated with the proportional length of the gene, a finding that has also been described for breast cancer [36]. Another interesting observation was that there was a significant enrichment of non-synonymous variants among cancerous tissue samples as compared with germline variants (p<0.001). This finding is in line with a recent observation of an accumulation of somatic missense mutations in cancer mitochondrial genomes [27, 36]. However, according to Ju et al. [27], the same mutational signature of the mitochondrial genome has been operative in the germline over evolutionary time and has therefore already acted on potentially synonymous sites, meaning that any new somatic changes are much less likely to be silent. Ju et al. concluded that despite the apparent high ratio of missense to silent mutations, the vast majority of mtDNA mutations were passengers with no convincing evidence for positive selection of missense mutations [27].

Half of the patients shared mutations between benign and cancerous tissue samples, yielding further support for the hypothesis that many putative somatic mutations are in fact low-level heteroplasmies undetected in the germline that have undergone clonal expansion in the tumor [28]. In addition, for three patients we analyzed distinct areas within the same tumor entity, because these areas differed from each other from a histopathologically perspective. Within each patient, the different tumor areas shared mutations with each other, but also exhibited mutations that were not found in other tumor areas. This discovery yielded further support for the clonal expansion hypothesis [28], but also underscored the impression that the mutations are acquired by the tumor itself [37]. Our observations on mtDNA mutations in oral cancer were similar to those made by a study on nuclear DNA mutations in paired benign and cancerous OSCC tissue samples: Izumchenko and colleagues reported that *NOTCH1* mutations were found in 54% of primary OSCC and 60% of pre-malignant lesions. Additionally, almost 60% of leukoplakia patients with mutated *NOTCH1* carried mutations that were also identified in OSCC, indicating an important role of these clonal events in the progression of early neoplasms [38].

Four out of six patients who developed a tumor recurrence showed mutations in the recurrence that had also been observed in the index tumor. This can on the one hand be explained by clonal expansion of low-level germline heteroplasmies as described before [28]. On the other hand also the "field cancerization" concept [39] could explain the phenomenon of recurring mutations in tumor relapse tissues [40]. This concept describes a pathogenic pathway for the development of multiple OSCCs arising in different areas of the oral cavity on the basis that OSCCs are often surrounded by genetically altered cells [41, 42]. In addition to our study, another recent study based on mtDNA sequencing of OSCC samples supported this hypothesis [43].

Finally, patients with tumor metastases in their lymph nodes exhibited–on the one hand–the same somatic mtDNA mutations in the primary tumor and in their lymph node metastases and–on the other hand–showed to a great extent an increase of mutant heteroplasmy from the primary tumor to the metastases, suggesting either a selection towards the mutated mitochondrial genome during tumor progression or a higher density of tumor cells in the lymph nodes.

Another interesting observation was that tumor samples showed a higher tendency to exhibit length heteroplasmy than their corresponding benign tissue samples. For non-coding regions such as the mitochondrial control region, these additional insertions in homopolymeric tracts (>4 bp) can be explained by a lack of accuracy of the polymerase gamma leading to slippage during replication and reflect the common mtDNA mutational pattern in tumor cells [44–47]. On the contrary, only one frame-shift causing length heteroplasmy in MT-ND5 was observed in two tumor samples. This is in line with the observation of greater constraints on mitochondrial genomes carrying protein-inactivating mutations. Cancer cells carrying such deleterious mutations are at a selective disadvantage and hence do not contribute to clonal expansions, underlining the importance of functional mitochondria to cancer cells [27].

In our previous study on prostate cancer, we found that the presence of somatic mutations in transfer RNAs (tRNAs) was associated with elevated PSA levels [14], and interestingly, mtDNA mutations in tRNA regions were also described to be correlated with tumor differentiation in OSCC [16]. In addition, the observation of an increase of the degree of heteroplasmy in a tumor that originated in the prostate gland and metastasized in the seminal vesicle [14] is in line with the present-study observation of an increase of mtDNA heteroplasmy from primary tumor to lymph node metastases. Moreover, the reappearance of heteroplasmies from primary oral carcinoma in recurrences has been described in mtDNA control region sequences [43], thus hinting to a common clonal origin of malignant cells. Taken together, our recent study improves the picture of previous mtDNA studies on OSCC [16, 43, 48–50] and breast cancer [36] and sheds more light on the complex mechanism of tumor development and differentiation.

In summary, our study has several strengths: (1) it targets the entire mitochondrial genome in various types of tissue from the same oral cancer patient over a median follow-up time of 30.5 month with both Sanger and NGS procedures; (2) the high coverage of sequence reads per base (~35,000) obtained with NGS allowed a detection of mtDNA heteroplasmy at the 1% level; and (3) the longitudinal design of our study targeting various types of tissue of the same oral cancer patient at several time points increases the power of our study. The small sample size (28 OSCC patients) and the fact that blood samples were not collected from all patients however could be considered as limitations of our study.

## Materials and Methods

### Ethics statement

This study was approved by the ethics committee of the Medical University of Innsbruck (study code UN3564).

### Patient data

Tissue samples were prospectively collected starting in June 2009. Patients aged 18 years or older, who had signed an informed consent, were consecutively included in the study. Patients with an oral squamous cell carcinoma that had been treated with chemotherapy or radiotherapy before surgery were excluded. The following clinical parameters were collected: age; sex; height; weight; smoking behavior; development of a tumor relapse during follow-up; development of metastasis; and TNM-stadium. Patients were followed for on median 30.5 months after tumor diagnosis (range 1–54 months).

### Tissue samples and DNA extraction

A pathologist determined benign and malign regions on HE-stained slides from fresh frozen tissue. Macrodissection was performed on consecutive slides (10μm slides with an average area of 10–15 cm^2^ of cancer and of benign tissue, respectively, which corresponded to ~5μg of DNA each). Then DNA was extracted from 85 tissue samples belonging to 28 patients with an EZ1 advanced Workstation with the EZ1 DNA tissue kit (QIAGEN, Hilden, Germany) and quantified with on a Tecan Infinity M200 with a Nano Quant Plate (Tecan Group Ltd. Männedorf, Switzerland). Tissue samples were taken from the primary tumors, the resection margins, the recurrences, the resection edges of the recurrences, blood, tumor areas in lymph nodes, benign areas in lymph nodes, from one second primary tumor and from one dysplasia (**S1 Table**).

### Sanger sequencing

All samples were Sanger sequenced using the high-quality protocol described in Kloss-Brandstätter et al. 2010 [14] with an average coverage of three to five sequence electropherograms per base pair. Electropherograms were aligned to the revised Cambridge Reference Sequence (rCRS; NC_012920) [24] with Sequencher (v5, GeneCodes, Ann Arbor, MI). Similar to a study on nuclear DNA mutations [23], the limit of detection for Sanger sequencing was identified at 10% of mutant mtDNA to the reference genome rCRS [24]. There, a nucleotide position was considered heteroplasmic, if a secondary peak of more than about 10% of the height of the primary peak was present. The exact proportion of a heteroplasmic mixture was extracted from each electropherograms covering the position of interest by a software component of Sequencher. Then, the mean value of the estimated proportions was calculated and this value was used for further analyses. Every mtDNA genome was evaluated independently by two well-experienced mtDNA technicians and validated by a senior mtDNA scientist with the mtDNA management software eCOMPAGT [51].

### Next-Generation Sequencing (NGS)

All samples were subjected to NGS at AROS Applied Biotechnology (Aarhus, Denmark) on an Illumina HiSeq 2500 with an average coverage of 35,000 reads per base pair. The mitochondrial genome was initially enriched by long-range PCR-amplification of two overlapping amplicons [14] and then quantified using the Quant-iT dsDNA Broad-Range Assay Kit on the Qubit Fluorometer (Life Technologies Corporation).

Indexed paired-end DNA libraries were prepared with the TruSeq DNA HT Sample Prep Kit (Illumina, Inc.). Therefore, amplicons were fragmented to 300 bp using Covaris Adaptive Focused Acoustics technology (COVARIS, Inc.) and purified with AMPure XP beads. After end repair, 3’-adenylation, and adapter ligation, DNA samples were enriched by PCR following the TruSeq DNA HT Sample Prep Kit protocol. The libraries were then quantified using KAPA Library Quantification Kits (Kapa Biosystems). All indexed DNA libraries were pooled together with equal molar ratios and were sequenced in a single lane of one flow cell on an Illumina HiSeq 2500 using 100-bp paired-end read chemistry.

The paired-end fastq files from the Illumina HiSeq 2500 were analysed with our highly parallelized in-house-pipeline based on Cloudgene ([52] and manuscript in preparation) called mtDNA-Server (http://mtdna-server.uibk.ac.at). In a first step the data quality was controlled by creating reports in order to verify values such as the “per base sequence quality”, “per base N content” or the “sequence length distribution”. In a second step the reads were aligned with BWA [53] to the rCRS. From the resulting BAM-files [54], the bases for each position relative to the rCRS were extracted, whereby only bases with a PHRED-score ≥ 30 and a mapping quality ≥ 30 were used for the heteroplasmy detection. After counting the frequencies of each nucleotide per position, only such ones were marked as heteroplasmies which exceed a certain threshold, which can be defined by the user. Having a mean coverage of over 35,000 throughout our samples after applying the filters, we set the detection limit for point heteroplasmies to 1%, meaning that a base was called heteroplasmic, if the minor component amounted to at least 1% for both forward and reverse reads separately and confirmed each other.

### Quality management and haplogroup determination

The mtDNA haplotypes were affiliated to haplogroups with HaploGrep [29] following Phylotree Build 16 [55, 56]. For quality assurance and in order to exclude contamination as potential source for the observed heteroplasmies, entire mtDNA profiles were generated from the oral surgeon, the pathologist and five lab technicians (Lab005 –Lab011) in addition to those four technicians (Lab001 –Lab004) genotyped for the prostate cancer study [14]. To do so, two profiles per sample where generated, each containing the shared variants (indicating no contamination happened), as well as the major and minor components of a heteroplasmic site respectively, as suggested by Avital et al. [57]. This way we could exclude or resequence samples, depending on the amount of remaining extracted mtDNA.

### Determination of the lower detection limit of heteroplasmies

In order to find the detection threshold for heteroplasmy, four samples with pre-defined mixtures of the DNA of two lab technicians were subjected to both Sanger and NGS. After exact quantification of the entire DNA content (including nuclear DNA), the samples were mixed according to the following ratios: 1+1; 1+9; 1+49 and 1+99. Then, the levels of heteroplasmy on the sites where the two lab members differed from each other where assessed with Sanger and next-generation sequencing (**S4 Table**).

### Statistical and bioinformatic methods

Pearson’s chi-square test was applied to 2x2 contingency tables. The Pearson product-moment correlation coefficient (“Pearson’s r”) was calculated as a measure of the correlation between two continuous variables.

In order to compare Sanger and next-generation sequencing technologies for the quantification of point heteroplasmy, the mixture ratios from Sanger electropherograms were exported with Sequencher and extracted with our in-house software from NGS reads. For all heteroplasmic positions, a Bland-Altman plot was generated for the comparison of the two different heteroplasmy measurements [26]. 139 heteroplasmic mutations were below the detection threshold of Sanger sequencing, and therefore there heteroplasmy values were set to the lower limit of detection divided by the square root of two, i.e. 10/1.414 = 7.07.

Each of the samples was represented on the graph by assigning the mean of the two measurements as the abscissa value, and the difference between the two values as the ordinate value. The mean difference reflected the estimated bias and was indicated as green line in the Bland-Altman plot. The standard deviation (SD) of the differences measured the random fluctuations around this mean. Therefore the 95% limits of agreement (average difference ± 1.96SD of the difference) were computed and plotted as red, dotted lines.

In order to identify a systematic difference between the measurements (i.e., a fixed bias), a one-sample t-test was applied to infer whether the mean value of the difference differed significantly from 0.

For comparing the frequencies of heteroplasmies at a certain level between benign and tumor samples, the numbers of heteroplasmies at a certain mixture level (e.g. 6.0–6.9%, 7.0–7.9%, etc.) were counted from all benign samples and from all tumor samples. We did not differentiate between heteroplasmies that were shared between benign and cancerous tissue samples and heteroplasmies, which occurred only in one kind of tissue. Instead, all heteroplasmic mutations were counted. Then, the frequency distributions from tumor samples and from benign samples were tested for equality with a chi-square test.

In order to evaluate the association between the occurrences of heteroplasmies in relation to gene size, the relative gene sizes were calculated by dividing the total number of base pairs per gene by the number of base pairs of the entire mitochondrial genome. Then, the number of heteroplasmies per gene was divided by the total number of heteroplasmies. Finally, these two numbers were tested for independency with a Pearson correlation analysis.

For the comparison of phylogenetic weights of heteroplasmic sites as estimated with HaploGrep [29] or EMMA [30] between different types of tissue samples, t-tests were applied. Statistical analyses were performed with IBM SPSS Statistics (version 22).

The immediate consequences (i.e. if the variants were synonymous of caused amino acid exchanges) of the observed mutations were assessed with MitoMaster [58] and MitImpact [59]. The conservation index (C.I.) was obtained from MitoTool [60]. The highest C.I. is 1, which means this site is completely conservative in 43 primate species. A C.I.-value of 0.651 indicates that 65.1% of 43 primate species have the same allele with the queried variant. MutPred [61] and PolyPhen-2 [62] were applied for predicting the functional consequences of non-synonymous mtDNA sequence variants. The number of reported NUMTs on each site was inferred from Li and colleagues [63]. In addition, the following bioinformatic tools were applied and the results collected via MitImpact [59]: FATHMM for the prediction of the functional effects of protein missense variants [64], PROVEAN to predict the functional effect of single or multiple amino acid substitutions, insertions and deletion [65], CAROL [66] Condel [67], PhyloP [68], and PhastCons [69].

## Supporting Information

S1 Fig Mutational distribution across the mitochondrial genome in oral cancer patients. (DOCX)

S1 Table Description of samples and analyzed tissue. (XLSX)

S2 Table Point and length heteroplasmic mtDNA mutations in different tissues from 28 oral cancer patients including bioinformatic predictions on pathogenicity and functionality. (XLSX)

S3 Table All heteroplasmic point mutations found with NGS before filtering; any interested reader is welcome to contact us for receiving the original raw data as FASTQ-files. (XLSX)

S4 Table Four different mixtures of mtDNA for validation purposes. (XLSX)

S5 Table Point heteroplasmic mtDNA mutations in 28 benign oral tissues. (XLSX)

S6 Table Point heteroplasmic mtDNA mutations in 28 oral cancer tissues. (XLSX)

S7 Table Comparison of two different NGS runs for sample MKG04_benign. (XLSX)
