# Supplementary material for: Validation of Next-Generation Sequencing of Entire Mitochondrial Genomes and the Diversity of Mitochondrial DNA Mutations in Oral Squamous Cell Carcinoma
Source: PLoS One. 2015 Aug 11;10(8):e0135643. doi: 10.1371/journal.pone.0135643 (PMC4532422; doi:10.1371/journal.pone.0135643)
Supplement: S1 Fig — (DOCX) [file pone.0135643.s001.docx]

**Supplementary Figure S1** Mutational distribution across the mitochondrial genome in oral cancer patients


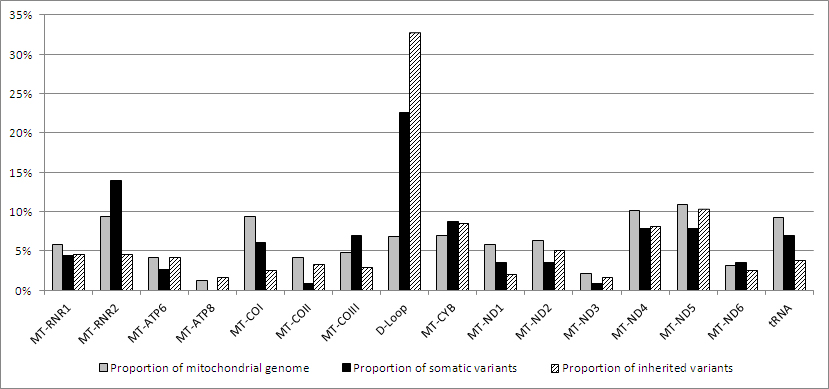


The grey bars reflect the proportion of the 16.6 kb mitochondrial genome that lies within each region/gene. The striped bars indicate the distribution of germline variants across the mitochondrial regions/genes. The black bars correspond to the distribution of somatic mutations across the mitochondrial regions/genes (D-Loop: 16024 – 16569; 1 – 576).
